# Bone morphogenetic proteins enhance an epithelial-mesenchymal transition in normal airway epithelial cells during restitution of a disrupted epithelium

**DOI:** 10.1186/1465-9921-14-36

**Published:** 2013-03-19

**Authors:** Natasha McCormack, Emer L Molloy, Shirley O’Dea

**Affiliations:** 1 Institute of Immunology, Biology Department, National University of Ireland Maynooth, Maynooth, Co. Kildare, Ireland

**Keywords:** Airway, Wound healing, Migration, BMP, EMT, Club cell (Clara)

## Abstract

**Background:**

Mechanisms of airway repair are poorly understood. It has been proposed that, following injury, progenitor populations such as club cells (Clara) become undifferentiated, proliferate and re-differentiate to re-epithelialise the airway. The exact phenotype of such cells during repair is unknown however. We hypothesised that airway epithelial cells (AECs) undergo some degree of epithelial-mesenchymal transition (EMT) in order to migrate over a denuded airway and effect re-epithelialisation. Furthermore, based on our previous findings that BMP signalling is an early event in AECs following injury *in vivo* and that BMP4 down-regulates E-cadherin expression and enhances migration in AECs *in vitro*, we hypothesised that BMPs could play a role in inducing such a phenotypic switch.

**Methods:**

Normal AECs were isolated from mouse lungs and analysed in a model of a disrupted epithelium. EMT marker expression and BMP signalling were examined by immunofluorescence, Western blotting and RT-PCR.

**Results:**

Following generation of a wound area, AECs at the wound edge migrated and acquired a mesenchymal-like morphology. E-cadherin expression was reduced in migrating cells while vimentin and α-smooth muscle actin (α-SMA) expression was increased. Re-expression of membrane E-cadherin was subsequently observed in some cells in the wound area following re-establishment of the monolayer. A transient increase in the incidence of nuclear phosphorylated Smad1/5/8 was observed in migrating cells compared with confluent cells, indicating active BMP signalling during migration. BMP antagonists noggin and gremlin inhibited cell migration, confirming the involvement of BMP signalling in migration and indicating autocrine signalling, possibly involving BMP7 or BMP4 which were expressed in AECs. Exogenous BMP2, BMP4 and BMP7 induced a mesenchymal-like morphology in AECs, enhanced the rate of cell migration and increased α-SMA protein expression in AECs.

**Conclusions:**

Following disruption of an intact epithelium, migrating AECs at the wound edge acquire an EMT-like phenotype involving altered expression of E-cadherin, vimentin and α-SMA. BMP signalling is involved in AEC migration and is likely to mediate the switch towards an EMT-like phenotype by altering protein expression to facilitate cell migration and wound closure. We propose therefore that acquisition of an EMT-like phenotype by AECs is a normal aspect of wound repair. Furthermore, we suggest that diseases involving fibrosis may arise because the EMT phase of repair is prolonged by chronic injury/inflammation, rather than being caused by it, as is the current paradigm.

## Background

Several cell types including basal cells and submucosal gland duct cells have been shown to function as stem/progenitor cells in proximal murine airways [1]. In distal murine airways, the abundant club cell (Clara) (previously known as the Clara cell) is believed to function as a ‘facultative progenitor cell’ that can contribute to re-epithelialisation of the airways after injury [2]. The club cell has been described as being functional flexible, or ‘plastic’, because it loses its characteristic differentiated phenotype while it is renewing the injured epithelium and has been described as ‘undifferentiated’ during this period. Subsequently, re-differentiation occurs and club cell functions are restored. However, the precise phenotype of reparative epithelial cells, such as club cells, while they are in the process of actively repairing tissues has received little attention to date. Such cells are poorly characterised in any tissue type, including the airways. During epithelial wound repair, surviving epithelial cells are required to become migratory in order to re-epithelialise damaged tissues. We have shown previously that an airway epithelial cell migration phase precedes a proliferation phase in the airway epithelium *in vivo* following acute lung injury and that increased bone morphogenetic protein (BMP) signalling is an early event during re-epithelialisation [3]. However, wound repair processes in the airways, and the signals that control them, remain poorly understood.

Myofibroblasts play a key role in tissue repair [4]. These are contractile cells and express α-smooth muscle actin (α-SMA). During repair, myofibroblasts secrete a temporary injury matrix of extracellular matrix (ECM) components. Epithelial cells migrate over the injury matrix and divide to replace damaged cells. During the remodelling stage, myofibroblasts promote contraction of the wound. While myofibroblasts play a key role in both normal and abnormal wound repair in the lungs, the source of these cells is unclear. It is thought that local mesenchymal cells, bone marrow progenitor cells and lung epithelial cells can all give rise to myofibroblasts, the latter through EMT [5]. EMT occurs during development and carcinogenesis and has been proposed as a contributory mechanism in fibrotic diseases [5]. During EMT, epithelial cells lose many of their characteristic properties and acquire features typical of mesenchymal cells. Protein expression is altered to allow cells to become less tightly attached to each other and more migratory. Transcriptional downregulation of epithelial cell-cell adhesion molecules occurs, and loss of E-cadherin in particular is considered a hallmark of EMT. Typically, cytokeratin intermediate filaments are replaced by vimentin and often α-SMA is expressed. In addition to development, cancer and fibrosis, an EMT-like process may occur during normal epithelial wound healing although this concept has been mentioned only sporadically in the literature [6].

Transforming growth factor-β1 (TGF-β1) is considered the prototype inducer of EMT and has been widely studied in this capacity [7,8]. Bone morphogenic proteins (BMPs) are highly conserved members of the TGF-β superfamily of cytokines and are involved in a number of processes throughout the body including morphogenesis, cell proliferation, differentiation, apoptosis and EMT [9]. BMP4 is an important regulator of lung morphogenesis during development and gremlin, a regulator of BMP signalling, negatively regulates BMP4 during lung branching morphogenesis [10,11]. However, few studies have addressed the role of BMP signalling in adult lungs in health, regeneration or disease. We have shown previously that increased bone morphogenetic protein (BMP) signalling is an early event in regenerating airway epithelial cells (AECs) *in vivo* following acute injury [5]. We have also shown that BMP4 down-regulates E-cadherin and stimulates migration of primary AECs *in vitro* indicating that BMP signalling may play a role in epithelial cell migration during normal wound healing in the airway epithelium.

Our group made the first report of an EMT process in AECs when we demonstrated that BMP4 induces EMT in human BEAS-2B cells [12]. Others have since shown that TGF-β1 induces EMT in primary AECs and that EMT may be enhanced in asthmatic airways [13,14]. Inflammation and elevated TGF-beta1 has been shown to result in dysregulated airway epithelial repair and fibrosis in a lung allograft via EMT [15]. Evidence of EMT in AECs in fibrotic lungs during obliterative bronchiolitis has also been reported [13]. However, EMT in pulmonary fibrosis remains controversial [16]. In the present study, we examined the phenotypic changes that airway epithelial cells undergo during wound healing in order to determine whether an EMT-like process, rather than de-differentiation *per se*, occurs. EMT has been reported in a study of human skin wound healing, with a role demonstrated for BMP2 [17]. This, along with our own data on the effects of BMPs on lung cells, led us to hypothesise that BMP ligands may be key regulators of this process. We report that AECs undergo an EMT-like process during wound repair, with downregulation of E-cadherin and increased expression of α-SMA and vimentin, and that BMP signalling plays a role in the process.

## Methods

### Primary AEC isolation

Ethical approval for this work involving animals was granted by the Biological Sciences Research Ethics Subcommittee, NUI Maynooth. Normal primary mouse AECs were harvested from female C57BL/6 J mice as described previously [18] with modifications described in [19]. In brief, lungs were removed from euthanized (sodium pentabarbitol overdose) mice, perfused with saline and digested with trypsin. Lungs were then chopped and filtered and suspensions were centrifuged at low speed to obtain clumps of airway epithelial cells. Because the cells are isolated in clumps, cell counting using a hemocytometer is not feasible. To ensure equal seeding densities, an aliquot of cell isolate was taken before seeding and an absorbance value (A450) was obtained using the Cell Titer 96 AQueous One Solution Cell Proliferation Assay (Promega Corp, Madison, Wisconsin, USA). Cell suspensions were diluted appropriately based on A450 values to obtain equal seeding densities.

### Wound closure assay

The wound closure assay is a modification of the Platypus Technologies Oris Cell Migration Assay (Platypus Technologies, Abingdon, UK). Each well of an Oris 96-well plate was prepared as follows: the centre area of the well was coated with 50 ng/ml fibronectin, allowed to dry for 2 hr and a stopper was then placed in the well to cover the fibronectin; the outer ring of the well was then coated with 50 ng/ml collagen and incubated overnight at 4°C; excess collagen was removed and primary mouse AECs were seeded in serum-containing medium (SCM) (1:1 Hams F12:M199 (Gibco, Glasgow, UK), 10% fetal bovine serum, 2 mM L-glutamine (Gibco), 100 U/ml penicillin and 100 μg/ml streptomycin (Gibco)) into this outer area. After 2 days, SCM was replaced with defined serum-free medium (SFM) (1:1 Hams F12:M199 (Gibco), 2 mM l-glutamine, 100 U/ml penicillin, 100 μg/ml streptomycin, 100 U/ml insulin-transferin-selenium (Gibco), 100 ng/ml hydrocortisone (Sigma-Aldrich, Dublin, Ireland) and 10 ng/ml epidermal growth factor (R&D Systems, Minneapolis, USA)). On day 5, plugs were removed and medium was replaced with fresh SCM. After 24 hr, cells were stained with 0.1% crystal violet stain (Sigma-Aldrich). The Oris ‘detection mask’ was used to visualize the wound area with a microscope. A photomicrograph of the entire wound area taken and from this, the total number of migrated cells in each wound was counted using Adobe Photoshop CS3 Extended Cell Count software. To achieve full epithelialisation of the wound area, plugs were removed at day 3 and cells were allowed to grow for a further 4 days in SCM.

### Treatment of AECs with BMP2, 4, 7, noggin and gremlin

Freshly isolated AECs were seeded onto Oris 96 well migration plates (Nunc, Roskilde, Denmark) in SCM. At day 2, medium was replaced with SFM. After 24 hr, supernatant was removed and fresh SFM was added containing appropriate concentrations of recombinant BMP2, 4 and 7 (Immunotools, Germany; R&D Systems, Minneapolis, USA) recombinant noggin (Peprotech, Rocky Hill, NJ, USA) or recombinant gremlin (Sigma-Aldrich).

### Immunofluorescence

Cultured cells were fixed with pre-chilled methanol. Tissue sections and cells were incubated with primary antibodies overnight at 4°C followed by appropriate Alexa Fluor® 488-labeled secondary antibodies (Molecular Probes, Invitrogen, Paisley, UK) for 30 min at room temperature. Nuclei were counterstained with DAPI (Sigma-Aldrich). Primary antibodies used were: α-SMA (Sigma-Aldrich), p-Smad1/5/8, (Cell Signalling Technology), E-cadherin (clone 36, BD Biosciences, Oxford, UK) and vimentin (Sigma-Aldrich). Secondary antibody only controls were carried out and representative images are shown in Additional file 1: Figure S1.

### RT-PCR

Semi quantitative RT-PCR for BMP2, BMP4 and BMP7 was carried out as described previously [6]. GAPDH was used as the housekeeper control. Primer sets are shown in Table 1.

**Table 1:** RT-PCR primer sets

***Primer***	***Sense***	***Antisense***
*BMP2*	5- CAGCATGTTTGGCCTGAAG −3	5- AAGTTCCTCCACGGCTTCTT-3
*BMP4*	5-CGTAGTCCCAAGCATCAC-3	5-ACAACATGGAAATGGCAC-3
*BMP7*	5- GGCTTCTCCTACCCCTACAA −3	5- GAACTCCCGATGGTGGTATC-3
*GAPDH*	5-CTGCACCACCAACTGCTTAG-3	5-CCAGGAAATGAGCTTGACAAA-3

### Western blotting

Western blotting and densitometric analyses were carried out as previously described [12]. Primary antibodies used were: actin (clone 20–33, Sigma-Aldrich) and α-SMA (Sigma-Aldrich).

## Results

### Induction of an EMT-like phenotype in AECs during restitution of a disrupted epithelium

**Figure 1:**
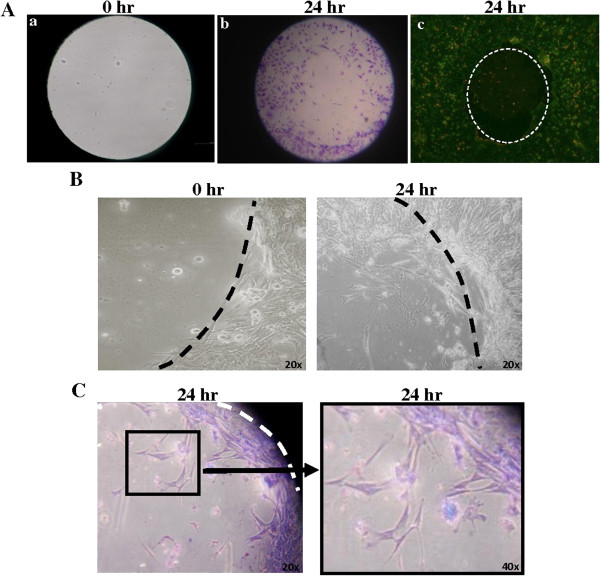
**AECs migrate and acquire a mesenchymal-like morphology in disrupted epithelium model. A.** AECs at 0 hr and 24 hr. (a) At 0 hr, no cells are observed in centre fibronectin-coated area where plug has been removed. Black area shows detection mask covering collagen coated area. (b) At 24 hr, cells have migrated onto the ‘wound’ area. (c) Cells stained with ethidium bromide/acridine orange to visualise the confluent outer area. White dashed line indicates area previously occupied by plug. **B.** AECs were allowed to become confluent around a central plug. The plug was then removed at 0 hr to mimic wound generation. AEC migration into the wound area was evident at 24 hr. **C.** AECs acquired a mesenchymal-like morphology while migrating into the wound area.

We have developed an *in vitro* model of wound healing that allows evaluation of the dynamics of airway cell morphology and migration during restoration of a disrupted epithelium. With this model we have attempted to recapitulate some of the key aspects of a wounded airway epithelium *in vivo*. The area in the centre of a well is coated with fibronectin, to represent a wound matrix, and a plug is placed on top of this. The remaining area encircling the plug is coated with collagen, to represent a more normal matrix, and AECs are seeded onto this area. The cells are cultured in serum-containing medium (SCM) for 2 days to become fully confluent and contact inhibited. To replicate the low rate of cell proliferation in an uninjured airway epithelium in vivo, the medium is replaced with serum-free medium for 3 days to allow the cells to become as quiescent as possible. Following this, the confluent monolayer is disrupted by removing the plug from the centre of the well. The cells are then free to migrate onto the exposed ‘wound’ area and their ability to restore a confluent monolayer is examined. A ‘mask’ which corresponds to the confluent area can be placed on the well when viewing under a microscope to distinguish the confluent area from the wound area (Figure 1A). We used this model previously to demonstrate that BMP4 enhances the migration rate of AECs [3]. Here, we used the model to examine the phenotype of AECs during migration and to further examine the role of BMP signalling in airway epithelial wound healing. We have shown previously that the method we use to isolate these murine AECs results in cultures that contain >80% club cells with the remainder comprising ciliated epithelial cells [19].

The epithelium was disrupted by removing a plug from the centre of a well containing a confluent monolayer of AECs. At 24 hr following plug removal, cells had migrated past the wound edge (Figure 1B). These migrating cells had an elongated mesenchymal-like morphology (Figure 1C). In contrast, cells in the confluent outer areas of the well, distant from the wound edge, retained a more regular epithelial-like morphology. In order to exclude the possibility the migrating cells were fibroblasts contaminating the epithelial cell preparation, cytokeratin expression was analysed in samples of cells prior to seeding and all cells were cytokeratin positive (data not shown).

**Figure 2:**
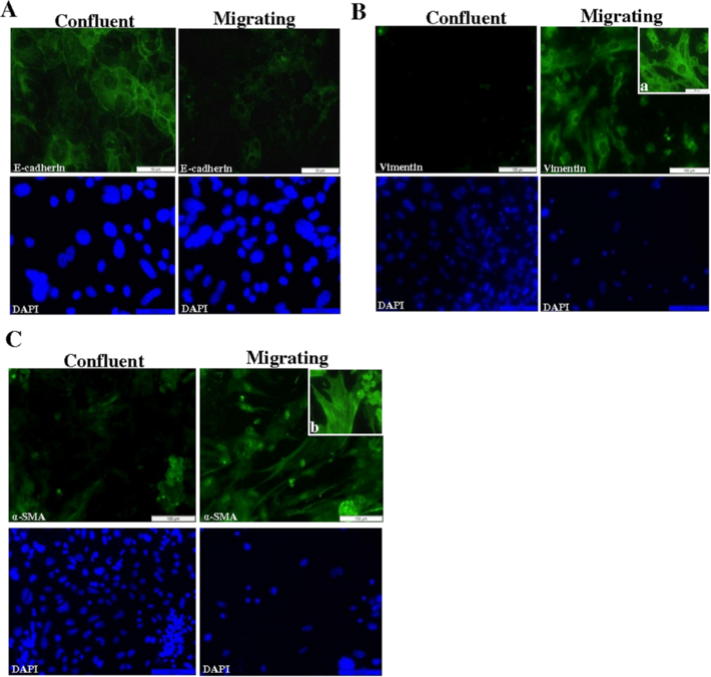
**AECs acquire an EMT-like phenotype in wound repair model. A.** Confluent areas of AECs retained E-cadherin membrane expression while E-cadherin expression was reduced in migrating AECs. In contrast, expression of mesenchymal markers **B.** vimentin and **C.** α-SMA was increased in migrating AECs. ‘a’ and ‘b’ show higher magnification. Respective Dapi nuclear counterstain images are shown.

We examined expression of EMT-related markers in migrating and confluent cells. Expression of the epithelial marker E-cadherin was reduced and more cytoplasmic in migrating AECs compared with confluent cells (Figure 2A). In contrast, expression of the mesenchymal markers, vimentin and α-SMA, was increased in migrating AECs with cells displaying the characteristic filamentous immunostaining pattern of these proteins (Figure 2B,C). Confluent undisrupted AECs displayed low level diffuse cytoplasmic immunostaining for both mesenchymal proteins, presumably as a result of the culture process.

**Figure 3:**
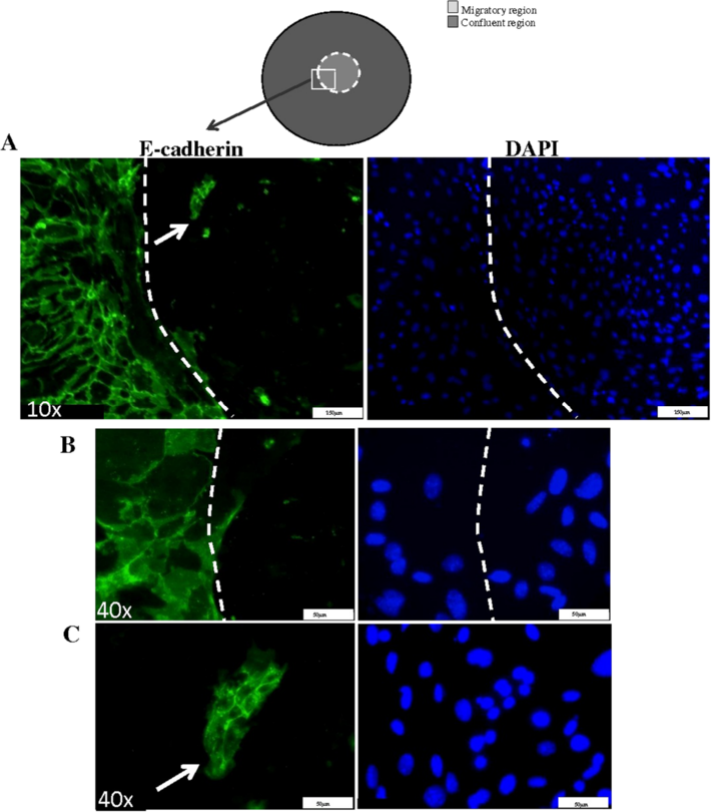
**E-cadherin expression in AECs that have completed migration at day 4 post plug removal. A.** Representative photomicrographs show membrane E-cadherin expression in stationary cells at the cell border (white dashed line). Migrating cells do not express membrane E-cadherin. White arrow indicated area where cells have re-expressed membrane E-cadherin. Respective DAPI nuclear counterstained images are also shown. **B.** Higher power immunofluorescence images of membrane E-cadherin in non-migrating stationary MAECs at the cell border. **C.** Higher power immunofluorescence images of re-established membrane E-cadherin expression following migration.

The monolayer was allowed to completely regenerate and the expression of E-cadherin and α-SMA was examined at day 4 post-plug removal, to determine if mesenchymal-epithelial transition (MET) could occur. Most cells remained α-SMA-positive at this stage (data not shown) but colonies of E-cadherin-positive cells were evident throughout the wound area (Figure 3). This suggests that some cells are capable of undergoing MET following restoration of cell-cell contacts. These primary AECs lose viability at the stage of the culture so longer time points could not be analysed to determine whether complete reversion to an epithelial phenotype was possible.

### BMP signalling during AEC wound healing

Given our previous findings that increased BMP signalling is an early event in AECs following acute injury *in vivo*[3], and that BMP4 down-regulates E-cadherin expression and increases migration in AECs *in vitro*[3,12], we further examined BMP signalling in the wound healing model. During BMP signalling, BMP ligands bind to cell surface BMP receptors. Activated receptors recruit and phosphorylate receptor-regulated Smads (R-Smads) 1, 5 and/or 8. Phosphorylated R-Smads form heteromeric complexes with Smad4 and translocate to the nucleus where they act as transcriptional co-modulators of specific target genes.

**Figure 4:**
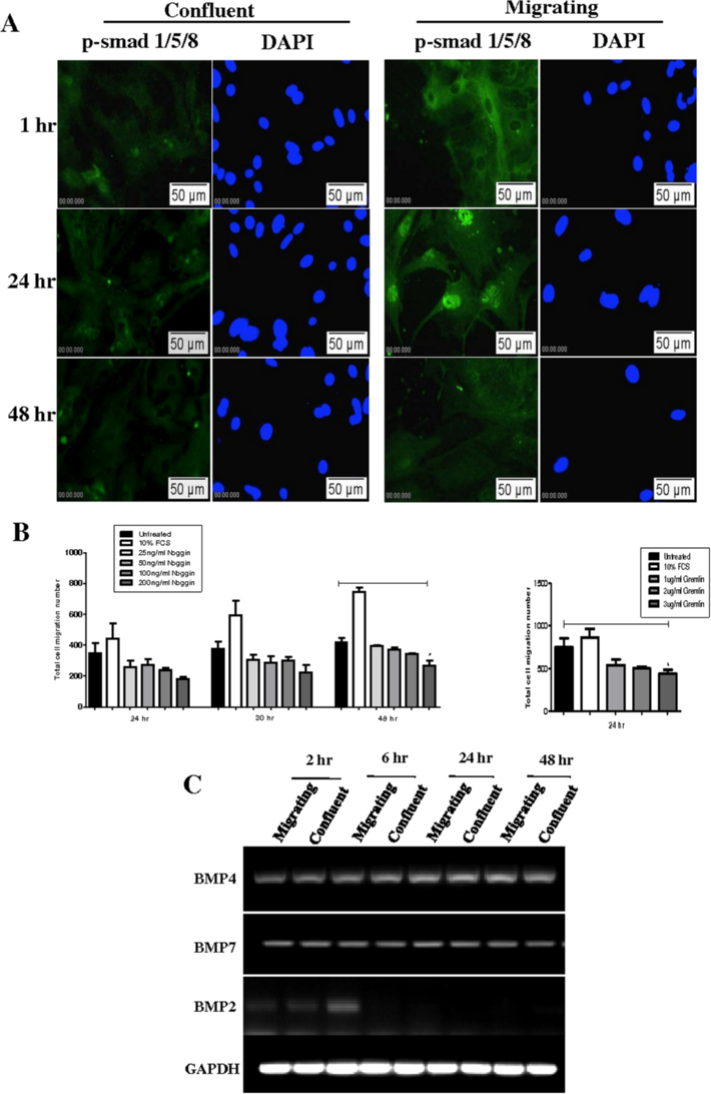
**BMP signalling in AECs in wound repair model. A.** Nuclear localised p-Smad1/5/8 was not evident in confluent AECs. In migrating AECs, nuclear p-Smad1/5/8 was detected at 24 hr post-plug removal but was not seen at 48 hr. **B.** Noggin and gremlin induced a dose responsive inhibition of AEC migration. Significance compared to control cells was obtained using the one-way ANOVA test (*P < 0.05), n = 3. **C.** BMP4 and BMP7 mRNA expression was detected in confluent and migrating AECs. BMP2 expression was low in AECs. Image is representative of 2 separate experiments.

We first examined whether the wound healing model reflected our observation of increased BMP signalling in AECs following injury *in vivo*. Immunofluorescence for phosphorylated Smad1/5/8 was performed on migrating and confluent AECs cultured in serum-containing medium at 1 hr, 24 hr and 48 hr following removal of plugs from the centre of wells containing confluent monolayers of AECs. Nuclear p-Smad1/5/8 was not observed in confluent AECs (Figure 4A). However, nuclear localised p-Smad1/5/8 was evident in migrating AECs at 24 hr and this was reduced again to baseline levels at 48 hr. This confirms that BMP signalling is activated in migrating AECs similar to our findings *in vivo*. Furthermore, it suggests that increased BMP signalling is a transient event during epithelial regeneration.

In order to determine if BMP signalling is required for AEC migration, BMP antagonists noggin and gremlin were used to inhibit BMP signalling during wound closure. Cells were cultured in serum-free medium prior to removal of plugs from the centre of wells containing confluent monolayers of AECs and noggin and gremlin were subsequently added in serum-free medium. Noggin induced a dose-responsive inhibition of the migration of AECs (Figure 4B). After 48 hr, cell migration rate was decreased 1.9-fold in the presence of 200 ng/ml noggin compared to untreated control cells. In order to exclude the possibility that noggin was reducing cell number rather than inhibiting cell migration, MTS cell viability assays were performed for all treatments at 24 hr and 48 hr and no significant difference between treatments was detected (data not shown). A second BMP antagonist, gremlin, also induced a dose-responsive reduction of AEC migration. After 24 hours of gremlin treatment at 3 μg/ml, cell migration rate decreased 1.9-fold compared to controls. The inhibitory effects of noggin and gremlin indicate that autocrine BMP signalling occurs during wound healing and induces migration in AEC.

As well as demonstrating that BMP signalling is required for AEC migration, these results also importantly indicate that autocrine BMP signalling in AECs plays a role in cell migration, as the assays were carried out in serum-free conditions in the absence of exogenous BMP ligands. We therefore examined BMP ligand mRNA expression in AECs. BMP2 is a homologue of BMP4 and both interact with the same BMP receptors as BMP7. Expression of BMP2, BMP4 and BMP7 mRNA was examined in migrating AECs at 2 hr, 6 hr, 24 hr and 48 hr post plug removal and also in confluent undisrupted AECs. BMP4 and BMP7 were constitutively expressed in confluent cells over the time course and no significant change in expression levels were observed in migrating AECs (Figure 4C). In contrast, BMP2 mRNA expression levels were low in confluent and migrating AECs suggesting that this ligand is less likely to be involved in autocrine signalling in AECs compared with BMP4 and BMP7.

### BMP2 and BMP7 induce an EMT-like response in airway epithelial cells

Given our previous finding that the rate of AEC migration in this wound healing model was increased in the presence of 100 ng/ml BMP4 at 24 hr, we examined if BMP7 and BMP2 had similar effects on morphology and migration of AECs.

**Figure 5:**
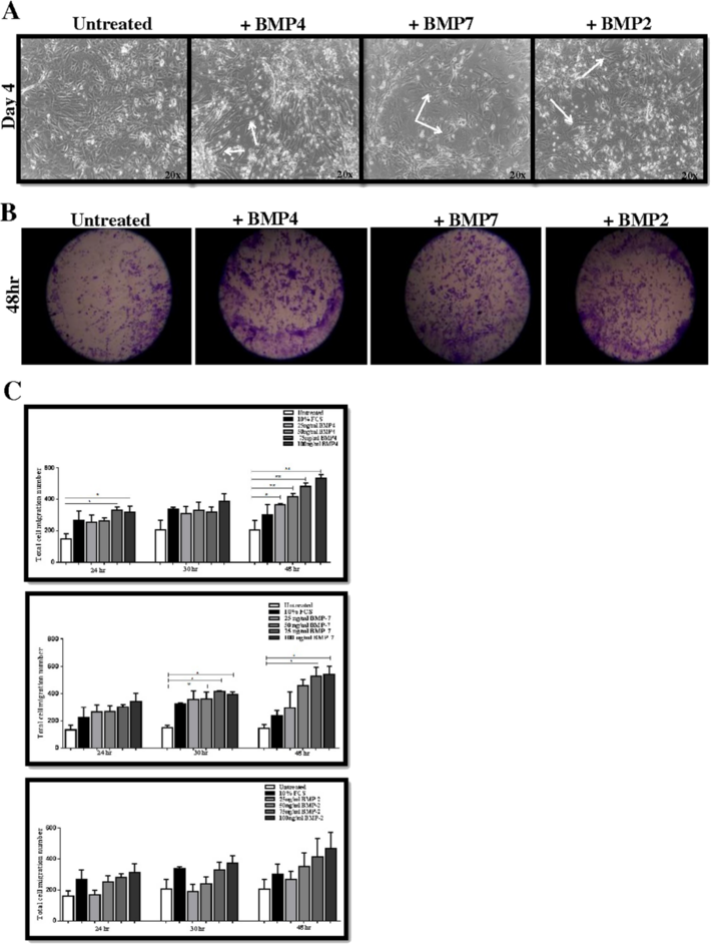
**BMP4, BMP7 and BMP2 induce a mesenchymal-like morphology and migration in AECs. A.** AECs cultured in the presence of BMP4, BMP7 and BMP2 acquired an irregular shape and were more scattered compared with untreated cells, magnification x20. **B.** Increased numbers of AECs were evident in the wound area at 48 hr in the presence of 100 ng/ml BMP4, BMP7 and BMP2, magnification x4. **C.** BMP4, BMP7 and BMP2 increased the rate of cells migration in a dose dependent manner. Significance compared to control cells was obtained using the one-way ANOVA test (*P < 0.05), n = 3.

Similar to our previous findings with BMP4, AECs cultured in the presence of BMP7 or BMP2 displayed an altered phenotype compared with control untreated cells. In the absence of exogenous BMP7, cells appeared tightly packed and had a regular, uniform morphology. In contrast, following treatment with BMP7, cells adopted an elongated, irregular shape and were more scattered (Figure 5A). A similar effect was seen with BMP2.

A dose-responsive increase in the rate of migration of AECs occurred in the presence of BMP7 over 24, 30 and 48 hr (Figure 5B,C). At 30 hr, migration rates were increased 2.7- and 2.6-fold in response to 75 and 100 ng/ml BMP7 compared to untreated control cells. After 48 hr, migration rates were increased 5.5-fold in response to 100 ng/ml BMP7. No significant increase in cell proliferation was detected (data not shown), indicating that cell migration, rather than proliferation, was occurring during this time. A dose-responsive increase in migration of AECs exposed to increasing concentrations of BMP2 was also observed over 24, 30 and 48 hr, although the extent of migration was slightly less compared with that induced by BMP7. An MTS cell viability assay was carried out with cells exposed to (100 ng/ml) BMP2 for 24 and 48 hr showed no significant increase in cell proliferation during this period (results not shown). Therefore, all three BMP ligands examined by us here and previously, BMP4, -7 and −2, are capable of inducing a mesenchymal-like morphology and increasing migration of AECs.

### BMP4, BMP7 and BMP2 increase expression of α-SMA in AECs

**Figure 6:**
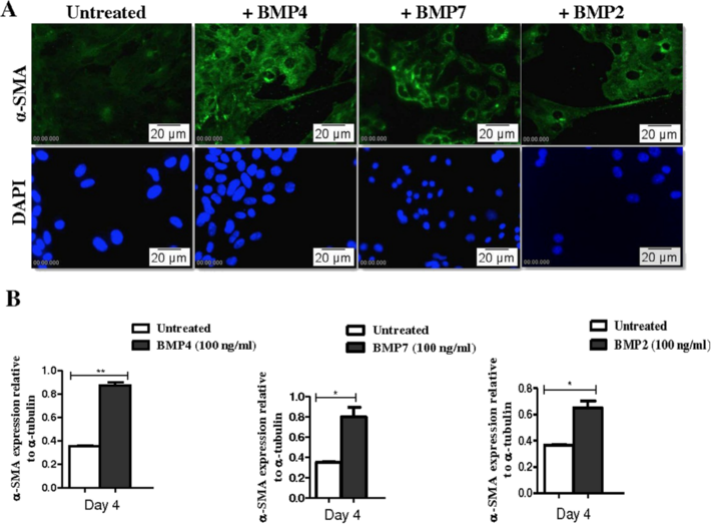
**BMPs increase expression of α-SMA in AECs. A.** Low levels of α-SMA were detected by immunofluorescence in control AECs that were not treated with exogenous BMPs. Increased levels of α-SMA were observed in cells cultured with BMP7, BMP2 or BMP4 compared with controls. Arrows show migrating cells with membranes extensions that contain characteristic α-SMA fibres. **B.** Densitometric analysis of Western blots also demonstrated that α-SMA levels were elevated in AECs cultured with BMP7, BMP2 or BMP4. Significance compared to control cells was obtained using the one-way ANOVA test (*P < 0.05), n = 3.

We hypothesised that the increased expression of mesenchymal proteins in migrating AECs was also due to BMP signalling. We therefore examined the effect of BMPs on α-SMA expression in AECs. Cells cultured in the presence of BMP4, -7 or −2 showed increased expression of α-SMA in AECs by immunofluorescence and Western blotting (Figure 6). Characteristic filamentous α-SMA immunostaining was evident in extensions of migrating cells.

## Discussion

The process of airway regeneration after injury and during disease remains poorly understood. In particular, the mechanisms underlying re-epithelialisation are unclear. In the field of airway stem and progenitor cell biology it is generally thought that, in murine bronchiolar regions, club cells function as a reparative cell population [6]. Following injury, these cells are believed to become ‘un-differentiated’ after which they proliferate and re-differentiate in order to re-epithelialise the airway wall. However, the precise phenotype of these ‘un-differentiated’ cells has not been characterised. Our data suggests that in fact, instead of being un-differentiated, these reparative cells temporarily acquire an EMT-like phenotype in order to carry out re-epithelialisation. This remains consistent with the reported loss of epithelial-related protein expression in these cells. We suggest that following re-epithelialisation of the damaged area, a mesenchymal-epithelial transition (MET) occurs to restore the differentiated epithelium.

In addition, we propose that BMPs play a key role in this wound-related EMT process, mirroring the important role of these proteins in lung development [20]. Several studies have identified a correlation between BMP signalling and EMT during embryonic development, fibrosis and cancer [21-23]. We now show that BMP induced EMT may also occur in airway regeneration processes. BMP2, BMP4 and BMP7 are important morphogens and are critical to a variety of developmental processes. BMP2 and BMP4 are 92% identical. Homozygous deletions of BMP2 and BMP4 in mice result in embryonic lethality involving abnormal heart development and a failure of mesoderm formation respectively, both involving EMT [24]. BMP7 also plays a key role in development [25]. While specific roles for BMP2 and BMP7 in lung development have not yet been described, BMP4 regulates airway branching and influences proximal-to-distal differentiation of airway epithelial cells in developing lungs [26]. Gremlin is expressed in pulmonary endothelial cells and has been shown to block BMP-stimulated wound healing in these cells [27]. The roles of these signalling molecules in adult lungs have not been widely studied however. We hypothesised that BMPs 2, 4 and 7 may play key roles in directing lung repair and regeneration after injury and we reported previously that BMP signalling is an early event *in vivo* in adult airway epithelial cells following acute lung injury [3]. We have also previously show that BMP4 induces morphological and phenotypic changes in AECs similar to EMT, including down regulation of E-cadherin, and that cell migration is also increased by BMP4 [3,12].

In the present study, we aimed to utilise an epithelial restoration assay to investigate specific aspects of BMP signalling in AECs during regeneration. The demonstration of nuclear localisation of p-Smad1/5/8 in migrating AECs *in vitro* here concurs with our previous finding of increased nuclear p-Smad1/5/8 in AECs *in vivo* in the early stages of regeneration after acute lung injury [3]. This activation of BMP signalling pathways was early and transient, both in our previous *in vivo* study and in the present study. We believe that BMP signalling is a key early event in regeneration, but is tightly controlled. The BMP signalling pathway can regulate itself by negative feedback control by directly transcriptionally upregulating inhibitor Smads 6 and 7 [28]. Chronic injury may overwhelm this negative feedback, leading to sustained Smad signalling and resulting in a more permanent EMT and ultimately fibrosis. In our studies, control wells with serum containing media showed enhanced cell migration compared with BMP ligands. Clearly factors such as fibroblast growth factors, FGF, TGF-β1 and possibly additional BMP ligands are likely to be present in serum and play a role in cell migration also.

A small number of other studies have examined BMP signalling pathway components in lung tissue. Smad activation has been reported in AECs during allergic inflammation [29,30]. However, the source of BMP ligands in the lungs, and the role of BMP-mediated signalling in AECs, has been unknown. We now demonstrate that autocrine BMP signalling occurs in AECs, possibly via BMP4 and BMP7 which we show are expressed in these cells, and that a function of this signalling is the enhancement of cell migration. Interestingly, levels of BMP2 mRNA were low, suggesting differential roles for BMP2 and BMP4 in AECs. We have also demonstrated that exogenous BMP2, BMP4 and BMP7 are capable of enhancing migration of AECs. Therefore, we can postulate that during airway regeneration *in vivo*, the source of BMP4 and BMP7 ligands is likely to include AECs themselves while other cell types are likely to be the source of BMP2. It has recently been reported that BMP2 is expressed in smooth muscle and vascular endothelial cells of blood vessels in vascular and skeletal tissues, but not by lymphatic vessels or macrophages [31]. Muscle and endothelial cells may also be sources of BMP2 in the lungs. Reports of the role of BMP7 in adult epithelial tissues have been somewhat contradictory. BMP7 has been reported to reverse TGF-β1-induced fibrotic effects *in vitro* in organs such as the heart, colon and kidneys [32]. In contrast, BMP7 does not reverse TGF-β1-induced EMT in human renal epithelial cells [33] and no inhibitory effect was observed in bleomycin-induced lung or skin fibrosis models [32,34]. Our data suggest that BMP7 promotes an EMT-like response in AECs.

In some circumstances, the wound repair process can become disregulated. During situations of chronic inflammation or recurrent injury, excess accumulation of ECM components at the site of injury can lead to permanently remodelled tissue. While myofibroblasts play a key role in both normal and abnormal wound repair in the lungs, the source of these cells is unclear. It is thought that local mesenchymal cells, bone marrow progenitor cells and lung epithelial cells can all give rise to myofibroblasts, the latter through EMT [4]. However, the extent to which each of these three cell populations contributes to myofibroblast recruitment, and whether these contributions differ in normal versus abnormal repair is unknown. Our data suggests that repairing AECs may be a source of these myofibroblasts. Furthermore we suggest that a paradigm in the fibrosis field could be adjusted also. It has been proposed that severe/chronic injury and/or inflammation in the airways and other tissues causes epithelial cells to undergo EMT that contributes to fibrosis [35,36]. Instead, we speculate that chronic/severe injury or inflammation may not directly induce EMT in epithelial cells to cause fibrosis. Rather, EMT occurs as a normal repair process during the initial injury but fails to reverse via MET because of the chronic injury conditions, leading to a permanent EMT that results in airway fibrosis. This is consistent with the view of fibrosis as a permanent injury state, but with the difference that the initial EMT event is a normal injury response. The problem may arise when the subsequent MET is delayed or inhibited by persistent injury/inflammation. If this is correct, strategies to treat fibrosis could address encouragement of MET. Further studies are required to fully characterise the extent to which EMT occurs in AECs during wound healing and to determine whether MET occurs once re-epithelialisation is complete.

## Conclusions

During wound repair, AECs acquire an EMT-like phenotype involving altered expression of E-cadherin, vimentin and α-SMA. BMP signalling is involved in AEC migration and is likely to mediate the switch towards an EMT-like phenotype by altering protein expression to facilitate cell migration and wound closure. We propose therefore that acquisition of an EMT-like phenotype by AECs is a normal aspect of wound repair. Furthermore, we suggest that diseases involving fibrosis may arise because the EMT phase of repair is prolonged by chronic injury/inflammation, rather than being caused by it, as is the current paradigm. In summary, this study indicates that regenerating AECs acquire an EMT-like phenotype during wound repair and that BMPs play a key role in regulating this process.

## Abbreviations

AEC: Airway epithelial cell; EMT: Epithelial-mesenchymal transition; BMP: Bone morphogenetic protein; α-SMA: α-Smooth muscle actin; SFM: Serum free medium; SCM: Serum containing medium; TGF-β: Transforming growth factor-β.

## Competing interests

The authors declare that they have no competing interests.

## Authors’ contributions

NMcC carried out the AEC isolations, developed the wound closure assay, carried out the migration and BMP assays and drafted the manuscript. EM designed and carried out the RT-PCR assays and analysis thereof. SOD conceived of the study, and participated in its design and coordination and helped to draft the manuscript. All authors read and approved the final manuscript.

## Supplementary Material

Additional file 1: Figure S1 Immunofluorescence images of secondary antibody controls. Antibodies are conjugated with Alexa 488 fluorophore. Images are shown for anti-mouse, anti-goat and anti-rabbit secondary antibodies. DAPI counter stain is also shown. Click here for file
