# Supplementary figures and images for: Bone morphogenetic proteins enhance an epithelial-mesenchymal transition in normal airway epithelial cells during restitution of a disrupted epithelium
Source: Respir Res. 2013 Mar 19;14(1):36. doi: 10.1186/1465-9921-14-36 (PMC3607850; doi:10.1186/1465-9921-14-36)

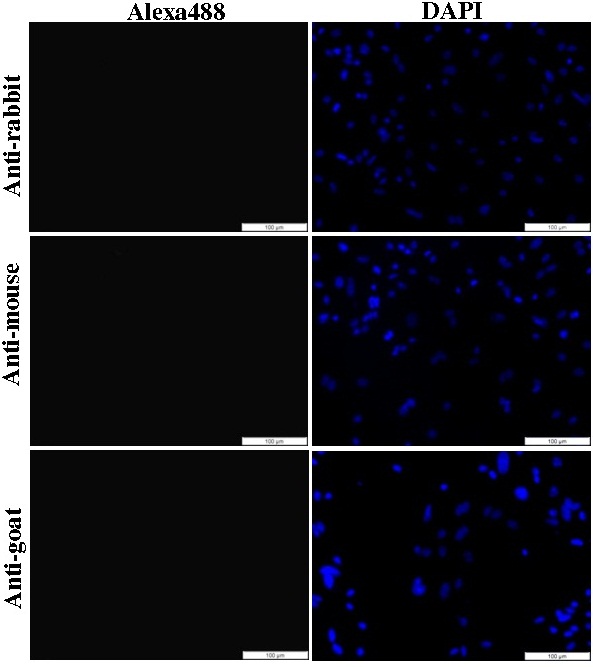

Supplement: Additional file 1: Figure S1 — Immunofluorescence images of secondary antibody controls. Antibodies are conjugated with Alexa 488 fluorophore. Images are shown for anti-mouse, anti-goat and anti-rabbit secondary antibodies. DAPI counter stain is also shown. [file 1465-9921-14-36-S1.jpeg]
